# Draft Genome Sequence of Perfluorooctane Acid-Degrading Bacterium *Pseudomonas parafulva* YAB-1

**DOI:** 10.1128/genomeA.00935-15

**Published:** 2015-09-03

**Authors:** Langbo Yi, Chongjian Tang, Qingjing Peng, Qingzhong Peng, Liyuan Chai

**Affiliations:** a School of Metallurgy and Environment, Central South University, Changsha, China; b College of Biology and Environmental Sciences, Jishou University, Jishou, Hunan, China

## Abstract

*Pseudomonas parafulva* YAB-1, isolated from perfluorinated compound-contaminated soil, has the ability to degrade perfluorooctane acid (PFOA) compound. Here, we report the draft genome sequence and annotation of the PFOA-degrading bacterium *P. parafulva* YAB-1. The data provide the basis to investigate the molecular mechanism of PFOA metabolism.

## GENOME ANNOUNCEMENT

Perfluorinated compounds (PFCs), such as perfluorooctane acid (PFOA) or perfluorooctane sulfonate (PFOS), have been widely used for the last 60 years in industrial, pharmaceutical, and commercial applications because of their strong surface-active effects and excellent physical and chemical stability (1, 2). These compounds have been found to be environmentally persistent, bioaccumulative, and potentially toxic, so environmental contamination has become a serious worldwide problem (3, 4). Biotransformation by microorganisms is one of the key remediation options that can be exploited to solve environmental pollution problems caused by these compounds. Our previous use of a microbial community enriched by PFOA from perfluorinated compound-contaminated soil in China led to successful isolation of a PFOA-degrading bacterium, *Pseudomonas parafulva* YAB-1. The strain YAB-1 has been deposited in the China Center for Type Culture Collection (CCTCC) under the accession number CCTCC M2015280.

To gain more insight into the bacterial degradation mechanisms of PFOA, the draft genome sequence of PFOA-degrading bacterium *P. parafulva* YAB-1 was determined. Whole-genome sequencing of strain YAB-1 was performed using a MiSeq (Illumina) system by generating paired-end libraries with an insert size of 400 bp and mate-paired libraries with an insert size of 5,000 bp. After filtering of the low-quality data, 1,419 Mb (270-fold coverage of the genome) and 408 Mb (80-fold coverage) of data were obtained, respectively. The paired-end reads were first *de novo* assembled using Newbler version 2.8 (Roche), and the scaffolds were connected according to 5-kb mate-paired relationships, then the gaps were filled using GapCloser with read mapping information. The coding sequences (CDS) were predicted using Glimmer version 3.0 (5). Homologous comparison of all of the genes was performed by BLAST with the NCBI nonredundant public database, KEGG, COG, Swiss-Prot, TrEMBL, and GO for function annotation. The tRNAs and rRNAs were identified using tRNAscan-SE and RNAmmer 1.2, respectively (6, 7). Other noncoding RNAs, including microRNA (miRNA), small RNA (sRNA), and small nuclear RNA (snRNA), were analyzed using Infernal software and Rfam database (8, 9).

We obtained 8 scaffolds consisting of 23 contigs with a total length of 5,119,844 bp, and the G+C content was determined to be 61.5%. From the genome composition analysis results, we found that the genome contained 4,807 genes with an average length of 937.8 bp, and the total length of genes was 4,508,235 bp, which makes up 88.1% of the genome. According to tRNAScan-SE and RNAmmer, we found 66 tRNAs with a total length of 5,153 bp, which makes up 0.10% of the genome. In addition, 2 rRNA operons and 67 other ncRNAs were also determined in the genome. A detailed comparison of the YAB-1 genome with the genome of other *Pseudomonas parafulva* strains may provide us with some insight into the molecular mechanism that allows for microbial degradation of PFOA.

### Nucleotide sequence accession numbers.

The draft genome sequence of *Pseudomonas parafulva* YAB-1 has been deposited at DDBJ/EMBL/GenBank under the accession no. LAWW00000000. The version described in this paper is version LAWW01000000.
